# Medical resilience and morality: a survey study on the opinions and actions of exemplary family physicians

**DOI:** 10.1186/s12875-021-01555-0

**Published:** 2021-10-25

**Authors:** Roger Ruiz Moral, Diana Monge Martín, Cristina Garcia de Leonardo, Santiago Alvarez Montero, Fernando Caballero Martínez

**Affiliations:** 1 Unidad de Educación Médica, Escuela de Medicina, Universidad Framcisco de Vitoria, Madrid, Spain; 2 Instituto Maimónides de Investigación Biomédica de Córdoba (IMIBIC), Córdoba University, Córdoba, Spain

**Keywords:** Burnout, Resilience, Professionalism, Medical values, Qualitative research, Medical ethics, Family medicine

## Abstract

**Background:**

Studies conducted to understand how family doctors develop resilience and deal with the challenges posed by work-related stress, usually have focused on identifying the elements that generate resilience from psychological perspectives and their impact on coping strategies. Few have explored the role that personal qualities and values that traditionally motivate family physicians can play as drivers of well-being and resilience.

**Objectives:**

To explore attributes that exemplary family physicians (EFP) consider important for their work and the elements that, for them, are source of gratification and resources in the face of the adversities they encounter in their practice.

**Methods:**

This is an exploratory study carried out by online survey. Eighty six doctors regarded as exemplary by their colleagues answered 7 close and 4 open-ended questions that explored their job satisfaction, the elements of their work that reward them, the difficulties and problems they usually encounter, the resources they use to cope with those problems, and the personal qualities they consider central to their work. Four researchers conducted a thematic (deductive and inductive) analysis of the free text responses received. Based on the results obtained, and through an iterative discussion process, the researchers proposed an integrated set of qualities at the core of their professionalism.

**Results:**

88.4% (76) of the doctors said they were satisfied with their work. However, they face problems (202 comments), including demanding patients, insensitive managers with unshared interests/care goals, excessive paperwork, work overload, or time pressures. Sources of gratification point to personal identity; clinical, relational, and collaborative efficiency; a holistic and comprehensive practice (centred on individuals); and a continuous search for excellence (149) and the root of their resources (135). These elements, in turn, corresponded to the attributes considered essential for the practice of family medicine (131).

**Conclusions:**

For EFPs, certain professional values give meaning to their clinical practice and are a source of well-being and resources. This central focus on professional values and qualities can help for better understand the burnout nature and expand the type of action that promotes resilience. Further studies using a less structured qualitative research will be needed to confirm/expand these results.

**Supplementary Information:**

The online version contains supplementary material available at 10.1186/s12875-021-01555-0.

## Introduction

Traditionally, certain values such as kindness, caring, good communication and interpersonal relationships, availability and accessibility, continuity of care, compassion, honesty, trust, and commitment to people, have been considered the essence of family medicine (FM). In these lies much of the value that society attaches to this specialty, and their relevance can be seen in the definition that WONCA Europe provides for this profession, where they are included as the cornerstones of clinical practice [1]. Some of these personal and specialty attributes have been related to patient satisfaction and positive health outcomes [2], and nowadays family doctors themselves stress their importance and their commitment to developing and respecting them [3, 4].

However, the practice of FM has changed a great deal in recent years. Most Western governments have pursued “neo-liberal” health policies [5, 6], commodifying the healthcare of many public health systems to make it an increasingly “competitive market” [7]. As a result, priority has been given to employee productivity and flexibility, which has led to an increase in job insecurity [8, 9], workload and unpaid overtime [10] and a reduction in the autonomy of the doctor [11], while doctors are expected to continue offering quality with fewer resources [8, 12]. At the same time, there have also been cultural changes, doctors in FM have seen an increase in patient demand and experience [9, 13], as well as new technologies for administering and evaluating the care they provide [11]. This has meant that family doctors have to cope with very demanding cognitive and emotional environments with high workloads and a great deal of dedication, and a large number of them are not satisfied, in particular with working conditions (remuneration, task management) but also with professional acceptance and social recognition [12, 14]. Burnout syndrome is a description (not a clinical diagnosis) of the degree of distress, emotional exhaustion, depersonalisation, and a sense of low personal achievement caused largely by these work-related stressors [15]. The numbers of doctors suffering from this in Europe vary from one in three to one in five, depending on the country [16].

In this hostile work environment, family doctors struggle to preserve professional attributes and values, and although many cope successfully with this situation [17], others perceive these as being inevitably eroded. The burnout can also be considered a physical and mental health response to this professional and personal “disappointment”; in certain situations, the doctor is unable to achieve or develop their mission or vocation as a professional [18]. This reflects a loss of the sense of being doctors, which reflects the kind of “moral injury” suffered by so many. The term “moral injury” comes from military psychology and refers to psychological trauma [19]. Moral injury occurs when there has been a betrayal of “what is right”, committed either by a person with legitimate authority (the profession), or by oneself; in a high-risk situation [20]. Moral injury can deteriorate character, affect confidence, increase despair, and heighten the risk of suicide and interpersonal violence, thus matching the effects described for medical burnout [15] and highlighting the practical significance of damaging one’s values.

Interest in understanding the factors that influence a physician’s well-being has increased in parallel with the recognition of increased physician burnout and its effects on the quality of the healthcare provided by resilient physicians [21]. Most studies have focused on identifying these factors from the psychological perspective and the impact they have on physical and mental indicators of burnout; however, very little attention has yet been paid to the ethical or “moral injury” incurred and the impact on a doctor’s attributes and values. This study intends to do so from a positive perspective, exploring the attributes or qualities that family physicians consider important today, and the role that these play in a practice that is healthy (effective and rewarding) for the doctors themselves. To this end, we have assumed that doctors who are considered exemplary by their colleagues are the repositories of these values and reflect them in their convictions and practice. Our intention is to carry out a first approach study to explore what these attributes or values are and to what extent they are a source of gratification and resources in the face of the adversities that these doctors typically encounter during the course of their work. This will allow us to gauge the validity and role of the attributes themselves as a source of gratification for the physician.

The objectives of this study were, therefore: 1) to identify the aspects that, in the opinion of doctors that are considered to be exemplary, a family doctor should cultivate so that their work is rewarding and effective; 2) to identify their sources of gratification in daily practice; 3) to detect the difficulties they experience and the nature of the resources they tend to use to confront these difficulties; and 4) as an additional objective, to identify those attributes and/or qualities detected in the professional profile of these physicians and to explore their coherence and relevance as useful indicators for developing resilience and clinical effectiveness in family doctors.

## Methods

This is considered as a preliminary study conducted by means of an online survey with close and open-ended questions.

### Sample

We sought out “exemplary physicians”, in other words, doctors widely recognised by their peers for their extensive knowledge, (“epistemic” experience) and/or the quality of their practice (“performative” experience) [22]. The following concept of “exemplary physician” was offered for the purposes of the study [23]: *“A clinical doctor who is a model for us or for trainees (residents and students), meaning that they are considered a professional worthy of imitation not only for their medical and technical knowledge but also for the quality of their clinical practice, and at the same time for their humanity in dealing with patients and families, their wisdom in general, and their constructive and collaborative spirit towards patients and colleagues*”. According to this definition, this type of physician may better reflect both the practical approaches and the values of family medicine than others. To select these doctors, using formal and informal networks, we identified a sample of 50 people responsible for training residents, representatives of residents and students and scientific society working groups and in specific geographical areas of the country (Madrid, Andalucía, Castilla, Basque Country and Galicia). They were asked to nominate the largest number of doctors in their sphere that they considered “exemplary” according to the criteria established. Using “snowball” sampling [24], we obtained a list of 102 doctors that included only those doctors who were nominated more than once. These doctors selected as “exemplary physicians” were considered as the purposive sample, they met the criteria of being currently active or having been active until recently (less than a year ago), with clinical practice being their main professional activity.

### Survey

A survey was used as a data collection method because the study was considered as piloting study, where to collect opinions from a high number of physicians about their present, most of them no sensitives, behaviours, was priorised.

Following principles about how design surveys [25, 26] four authors designed a preliminary survey with eight closed questions to obtain quantitative information related to the clinical context, experience, workload and other non-healthcare responsibilities of the respondents as well as their job satisfaction perception. To explore aspects of job dissatisfaction, strategies used to cope with them and essential features of the work of a family doctor, three open questions were also designed. In order to establish definitive content and response process validity a previous pilot testing study was carried out in a convenience sample made up of primary care physicians and hospitals in the authors’ environment [27]. The survey was finally sent online to 76 physicians from Madrid and surroundings, with the additional request to evaluate technical aspects of the survey itself. These were family physicians well known and appreciate for their clinical work and responsabilities but not strictly considered as outstanding physicians as we have defined in this study. A response was obtained from 51 doctors with 23 technical comments on the survey and its different items. Based on these comments and their results, the survey was reworked. Most of the items were rewording and one item, originally close-ended, was finally drafted as a free text question. A final 11-question survey was prepared, involving seven closed questions, with different answer options and four open questions (Additional file 1). Administration details: The survey was sent through an online platform to all those selected. Previously a personal letter was sent to all doctors chosen asking for their consent and explained the main objectives of the study, how much time they should given to complete it and clarifying that all responses would be treated anonymously. A period of 3 weeks was stablished for responding with three remainders sent.

### Data analysis

The quantitative data were subjected to a descriptive statistical analysis. For the analysis of the responses to the free text questions, a qualitative thematic analysis was carried out. The first phase of this qualitative analysis included a deductive approach that established 14 (5;5;4) main predetermined categories from the existing literature and identified in the results of the pilot study (Table 1).

**Table 1:** Predeterminated themes for analysis

Rewarding elements	Dissatisfaction elements	Resources for solving difficulties
Service/Helping others Doctor-patient relationship Whole person care Teamwork Others/Teaching-researching	Work overload Administrative burden Management/Organizational issues Patients Teamwork/colleges	Management/Organizational Selfcare activities Teamwork strategies Relationships

Subsequently, from the free text, the narratives that provided relevant information related to the main categories were identified. In these narratives, codes and subcodes were identified [28], which were quantified to describe which main categories appeared more frequently in the narrative responses of the participants. Considering their relevance, some of these new codes were considered as “new main categories”. This deductive and inductive quantification and classification of codes to construct and justify the categories can be carried out in qualitative designs [29], and has been used in health sciences. In this study we have included the codes (and narrative fragments) that provided relevant information with the main categories.

**Fig. 1:**
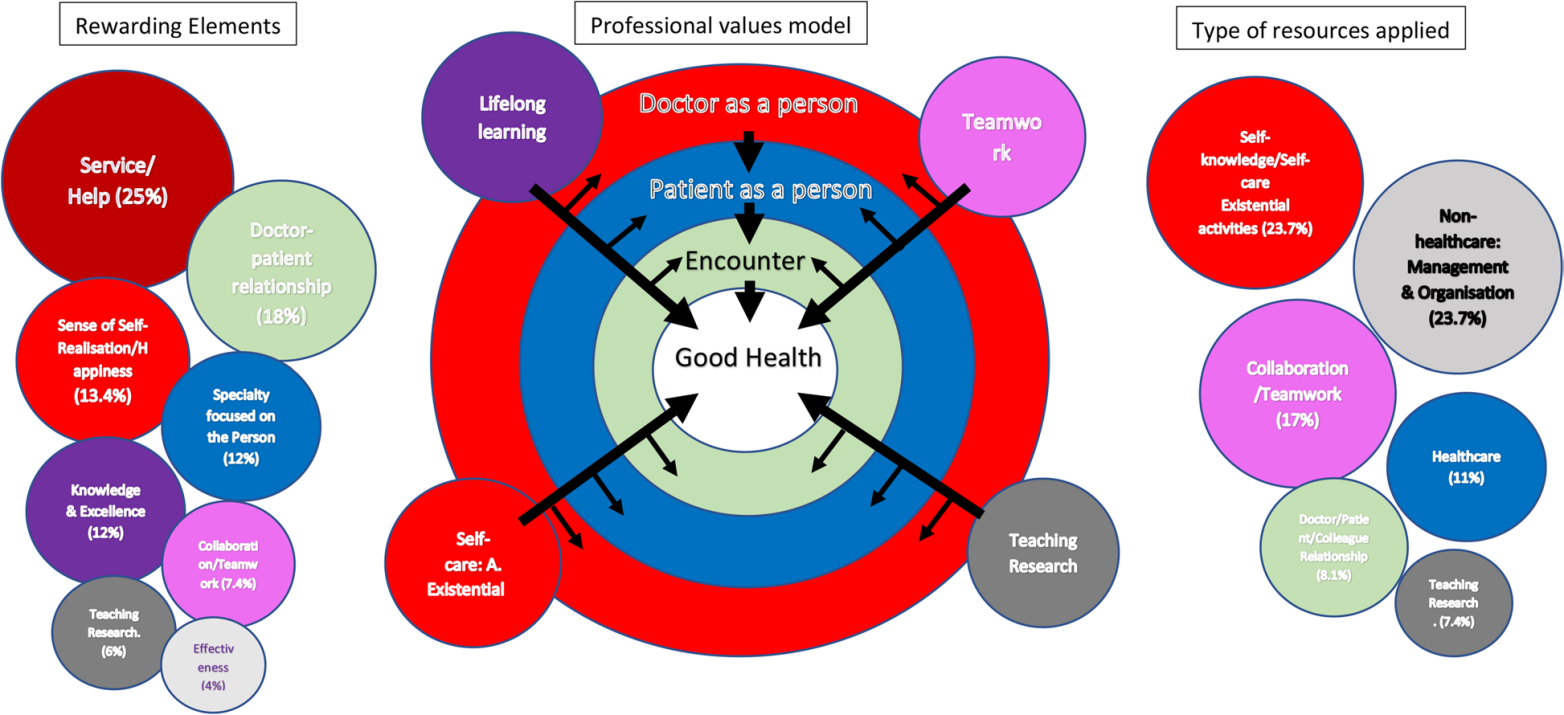
Integrated model of the personal values required to be a good family physician (central circles)

Each of the four authors (RRM, SAM, DMM, and CGL) contributed their own codes and subcodes in the transcripts of the response content, with arguments about their interpretations in the most ambiguous points. A discussion process was undertaken supporting and challenging each others reflexivity and assumptions. The key findings are reported in this document. Here we present the strongest emerging themes, using the quotes that best illustrate the shared meaning. For the set of categories identified as essential qualities for a family physician (question 11), each researcher developed their own integrative model that was also discussed in an iterative process that considered existing literature and previous conceptual related theories and frameworks. The model offered in this paper illustrates the consensus and represent a tentative approach (Fig. 1).

## Results

Of the 102 doctors contacted, 86 (84.3%) replied. Of those, 88.4% (76) worked in Primary Care and 11.6% were from Emergency Services (10); 72% (62) had more than 20 years of work experience and 25.6% (22) had between 16 and 20 years. 77.9% (67) of the doctors had, in addition to their clinical duties, other responsibilities, principally teaching students and residents and administrative duties in committees or working groups; 16.3% (14) had additional non-medical responsibilities. 91.9% (79) had family responsibilities. 53.5% (46) of the respondents saw an average of 31-40 patients per day, 16.3% (14) saw between 21 and 30 patients, and 15.1% (13) attended more than 40 patients every day.

88.4% (76) of the physicians stated that they were satisfied with their work both personally and professionally, only 3 (3.5%) felt unsatisfied, and 7 (8.1%) were not sure how to respond. Additionally, they were asked if their work was a source of satisfaction and whether they enjoyed it: just over half EFPs (45; 52.3%) stated that they always or almost always enjoyed it, 36 EFPs (41.9%) said they typically enjoyed it, one doctor (1.2%) only enjoyed it sporadically, and 4 (4.6%) stated that they “suffered” from their work, which was a burden.

Tables 2, 3, 4 and 5 list the categories and subcategories identified with examples of the most representative statements received regarding the main reasons why EFPs enjoy their work (the most satisfactory aspects) (question 8; 149 comments), the causes of dissatisfaction or distaste with their work (question 9; 202 comments), the resources that these EFPs routinely employ to attempt to address these difficulties (question 10; 135 comments), and the elements they consider important for effective and rewarding clinical performance (referred to in question 11 as ‘advice to beginners’; 131 comments), respectively.

**Table 2:** Elements of their clinical performance that exemplary physicians report as rewarding

Main category	Subcategory	Example(s)
1) Service/Help 25% (37)	To patients To colleagues To trainees	*“Being helpful to patients, residents, students and sometimes even co-workers.”*
2) Doctor-patient relationship 18% (27)	With patients With colleagues With trainees	*“I love dealing with patients and the good therapeutic and personal relationships I have established over the years”;* *“The trust that is established makes it possible to maintain a relationship that goes beyond mere healthcare.”*
3) Knowledge and excellence seeking 12% (18)		*“Constant learning with each problem.”*
4) Speciality Characteristics and Focus on the Person 8% (12)	Comprehensive care of an individual Healing vs. curing Continuity Longitudinal Universal Polyvalence	*“Family medicine is about caring for people, and that’s why it’s the nicest by far”;* *“Helping to improve people’s quality of life and supporting them even though we don’t cure them most of the time”;* *“Polyvalence: not knowing what is going to come through the door and the possibility of developing very different skills to deal with the various health problems”;* *“I make health care universal.”*
5) Collaboration/Teamwork 7.4% (11)		*“I am enriched through the experiences that my colleagues share.”*
6) Self-fulfilment/Meaning 6.7% (10)		*“I see meaning in what I do, even if we don’t cure people”;* *“It allows me to fulfil myself personally and professionally.”*
7) Happiness 6.7% (10)		*“I simply enjoy what I do and I can’t tell you why.”*
8) Teaching 6% (9)		*“I enjoy teaching my residents.”*
9) Sense of effectiveness 4% (6)		*“It’s the feeling of a job well done”;* *“I enjoy ‘good’ activity, a well done Medical History, for example. I could go on, with many things we do well every day.”*
10) Other 6% (9)	Recognition Material aspects Applying specific techniques Physical environment	*“I feel useful to society because I feel the recognition of my colleagues, the recognition of society* *and* *because I feel that the institution where I work understands my problems”;* *“I find certain procedures stimulating, like ultrasounds, dermatoscopy, and so on”;* *“It gives me enough money to have a good quality of life”*

**Table 3:** Main difficulties or aspects of regular work that generate dissatisfaction for exemplary doctors

Main category	Subcategory	Example(s)
1) Healthcare problems (64) 31.6%	Excessive number of patient visits Insufficient time for care	*“I have to see more patients a day than I am able to do satisfactorily”;* *“The lack of time to analyse patients properly.”*
2) The company/healthcare management (32) 15.8%	Administrative pressure (re. tasks, objectives) Management behaviour (arrogance) Difficult to achieve goals Interest only in the quantitative at the expense of the qualitative/human No/little autonomy for the doctor	*“Pressure from management to fill out forms and protocols to achieve measurable goals”;* *“Managers at the same level as us treat us arrogantly”;* *“Unrealistic goals, in many cases not adapted to the patient”;* *“Only financial results matter, not qualitative ones, let alone the human ones”;* *“Lack of autonomy to manage your own timetable.”*
3) Excessive bureaucratic burden (20) 9.9%	Administrative tasks Multiple tasks to be performed at the same time Management of sick leave Priority given to protocols and records that are of little use for the patient	*“Doing administrative or registration work with little healthcare value”;* *“Having to perform multiple tasks simultaneously”;* *“I don’t like the paperwork involved in sick leave”;* *“The obligation to fill out forms and protocols to achieve measurable goals.”*
4) Relationship with colleagues & team (19) 9.4%	Poor coordination with nursing Impossibility of choosing team Burnt out colleagues Colleagues at the hospital care level	*“Poor collaboration with nursing staff”;* *“Not being able to choose your own team, it’s imposed on you”;* *“Some colleagues are toxic”;* *“The relationship with some fellow specialists is overly depersonalised, bureaucratised and distant.”*
5) Relationship with a certain type of patients (17) 8.4%	With a culture of immediacy No health education With trivial conditions No appointment With certain behaviour (demanding, not very empathetic, etc.)	*“Patients who have a culture of immediacy”;* *“Poor health education of some patients”;* *“Visits involving no/trivial conditions”;* *“Patients who come in again and again without an appointment”;* *“The few welfare patients who fake symptoms so they can get, and stay on, sick leave.”*
6) No/little recognition (14) 6.9%	In general, towards the specialty By management regarding the doctor’s work	*“Little appreciation of family medicine by healthcare, academic (university) and political institutions”;* *“The lack of recognition and compensation for those doctors who work well.”*
7) Work organisation (12) 5.9%	Slow response to certain problems Lack of flexibility Software (ICTS) Specific organisational aspects	*“Sometimes solving a patient’s problem takes forever due to bureaucracy”;* *“Difficulty in solving incidents external to our team due to lack of flexibility”;* *“Computer-related aspects can become hopeless.”*
8) Pay (8) 3.9%		*“Inadequate pay especially for being on call.”*
9) Scarcity of resources (7) 3.4%	Physical spaces Human resources	*“Physical space is very precarious”;* *“Lack of personnel, especially for covering things like holidays.”*
10) Other (9) 4.4%	Research and teaching (scarce) Routine Low resolution capacity Social problems Call Pharmaceutical industry relationship	*“Research must be done outside working hours”;* *“Low priority and resources for other activities (teaching, research)”;* *“The routine sometimes exasperates me”;* *“I don’t like the relationship with the pharmaceutical industry.”*

**Table 4:** Type of resources or strategies applied by exemplary family physicians to resolve difficulties

Category	Subcategory	Example(s)
1) Non-healthcare: Management & Organisation (32) 23.7%	Specific measures for managing activities Working together with managers Specific activism measures Involving patients, managers and specialists together Participation in commissions from management	*“I limit the use of protocols when I find it unrealistic to follow them”;* *“I schedule all family business for the 1 day I work in the morning”;* *“I schedule home-visits“;* *“I have one diary for what is important and another for the ‘little things’”;* *“I allocate two slots for patients with complex medical issues”;* *“I have advocated strategies for self-care and the recovery of professional dignity and have agreed with management for these to be included in the centre’s activities”;* *“I’ve worked with neighbourhood associations on specific issues”;* *“When a serious problem arises, we include the affected patients in a discussion on the solutions; the managers are in favour of this”;* *“I collaborate on commissions encouraged by management: Pharmacy,* etc.*”*
2) Self-care, self-knowledge and “existential” activities (32) 23.7%	“Friendly” physical spaces Use of humour Adjusting expectations/putting things in perspective Specific mindfulness and self-care strategies at work Spaces for reflection Doing other extra-professional activities Encouraging positive thinking	*“I decorate the office in a bright way and change it up periodically”;* *“I try to smile all the time... and it works!”;* *“I try to have fun with the patients, I use humour a lot”;* *“I take short breaks between patients. It only takes a few seconds to look at the next patient’s medical history and become aware of myself”;* *“Sometimes, when I’m very tired, I close the door and relax for a few minutes”; “I usually set aside time for reflection/talking/having fun with a colleague who is free”;* *“Physical exercise. Relaxation exercises. Relaxing with family and friends in my free time, unwinding”;* *“I do other activities: theatre, literature,* etc.*, that help me put everything in perspective”;* *“I usually look ahead and think of the satisfaction I get from helping the patient with their problem and trying to resolve it or at least make it better.”*
3) Teamwork /Collaboration (23) 17%	Encouraging solidarity and shared experiences between team members Task delegation strategies Specific nursing strategies Working with social services Specific strategies with specialist colleagues	*“I try to enjoy the shared moments with the team”;* *“I share my experiences (critical incidents) with my colleagues”;* *“I collaborate in working groups with colleagues on specific aspects for improving healthcare”;* *“By coming to agreements with my staff, I have been able to delegate many actions”;* *“Participation in multidisciplinary groups with specialists, nurses,* etc.*”;* *“On the team, we’ve included social services in many decisions”;*
4) Healthcare (15) 11%	Optimising Primary healthcare strategies: longitudinality; education, health,... Joint care with nursing Focus on problem-solving in healthcare Encouraging “complete care” Use of new technologies (ultrasound)	*“I use my relationship with and knowledge of my patients for many things,* e.g.*, if I’m overloaded 1 day I ask them directly if they can wait until another day”;* *“Training and supporting nurses to carry out self-limited minor processes”;* *“Supporting triage operations in appointments to assess whether a doctor or nurse appointment is necessary and prioritising these accordingly”;* *“Being ‘present’ at each point, I can forget about the superfluous”;* *“Focusing on the care of the patient I’m treating and not thinking about the waiting list, delays, absent colleagues, emergencies...”;* *“With the incorporation of ultrasound I have gained both in decisiveness and in relation to my patients.”*
5) Doctor/Patient/Colleague relationship/ (11) 8.1%	Trust Actively empathising Being assertive Prioritising respect and kindness	*“I always try to improve the trust my patients have on me”;* *“I consciously strive to empathise and try to understand other points of view”;* *“I avoid conflict with conflicting people”;* *“I’m assertive but always respectful.”*
6) Non-healthcare: Teaching, Research (10) 7.4%	Participating in new projects Teaching residents and students Ongoing education Research	*“I get involved in other projects: research, publications, conferences, teaching... I like it and it lets me escape somewhat from the pressure of the clinic”;* *“Training, teaching residents, community activity.”*
7) Low pay (2) 1.5%	Adapting to income Moderating material desires	*“Throughout life you adapt, it’s about wanting less, about what you are more than what you have.”*

**Table 5:** Personal attributes that exemplary family physicians believe support effective and rewarding clinical practice in family medicine

Category	Subcategory	Example(s)
1) The doctor as a person Self-knowledge Self-care (33) 25%	Personal reflection Critical and flexible thinking Accepting limits Managing emotions Presence Authenticity Encouraging certain attitudes	*“Taking time to get to know yourself in depth from the start, and at the same time taking care of yourself for a long journey”;* *“Learning about and reflecting on yourself and your role as a family physician”;* *“We must learn not to be rigid about anything. No truth is absolute”;* *“Knowing how to accept our limits and not blaming ourselves if we do something wrong (we are also people)”;* *“Learning to manage our emotions so we do not harm the clinical relationship”;* *“It’s about arriving at work, leaving your problems outside the office and making the patient see that you care about them and want to help them. To do this, you must try to be very present in the office”;* *“You have to discover your own style and work on it, and be yourself in what you do”;* *“Be curious in every encounter.”*
2) The Patient as a Person (23) 17.5%	Dimensions of the person Context of the person The doctor as a person	*“Don’t look at patients as objects of study and a source of learning, but as people, and try to understand them so that we can help them”;* *“Never forget that you will be working with people and that their ailments and illnesses occur in a personal, family and social context that is very important to understand and take into account”;* *“See the person, with their values and circumstances, behind every clinical problem”;* *“Treat each patient as we would like to be treated ourselves.”*
3) 5) Doctor-Patient relationship (20) 15.2%	The relationship The support The “medical friendship”	*“Realise that the core of our work involves communication and relationships”;* *“Be compassionate and try not to judge patients by trying to support them even if we can’t cure them”;* *“Always try to empathise with the patients because that will create a ‘friendship’ that will also help us when we have difficult moments at work.”*
4) Positivity/Effectiveness (14) 10.7%		*“Try to ‘always’ get something positive out of the day, something beneficial and comforting, because there is always something;* *“It’s always essential to be efficient in your work, and maintain this.”*
5) Service/Help (14) 10.7%	Usefulness to others Vocation	*“We mustn’t lose our goals, our vision, those reasons that led us to study a humanistic career like medicine, to be at the service of people”;* *“Don’t lose your vocation to serve.”*
6) Ongoing learning and Updating (13) 9.9%	Skills Knowledge	*“Don’t give up on learning new skills and incorporate these to resolve new patient problems”;* *“You must never stop studying and updating yourself.”*
7) The doctor as a person Activities with existential value (12) 9%	Recreational Meditative Relational	*“Read literature, essays, poetry, cultivate friends, make love, eat well and do sport”;* *“Learn not to take work home, to disconnect, fill life with other things: hobbies, family...”;* *“Meditate every day.”*
8) Collaboration/Teamwork (10) 7.6%		*“Always try to find a time to interact with your co-workers”;* *“Treat your fellow professionals well, like your brothers”;* *“Cultivate a good atmosphere in the team because you will reap the rewards.”*
9) Teaching/Research (4) 3%		*“Get involved in teaching and research.”*

Although some responses were briefs with sentences with few words there were many providing copious narrative feedback with high conceptual richness. Among the rewarding aspects, category number 4 includes a sense of holism and integrality that defines the person and seems to be inherent to the specialty and its characteristics; the answers referring to “healing” as something related to those concepts have been included here. Finally, the category “Happiness” (number 7), which includes generic statements of satisfaction, was kept separate. However, to develop the final scheme, it was included in the category “Self-fulfilment/meaning”. In the four questions, but especially in those exploring the type of resources used, the answers ranged from generic statements (e.g., *“delegate as much as possible”* or *“deal with situations using constructive criticism”, “apply self-care strategies”)*, to very concrete strategies (e.g., “*Working with the ‘triap’ [Primary Care Triage] model often allows me to address the lack of management involvement in primary care”*, *“If I’m too tired, I’ll close the door and relax for* a *few minutes or go downstairs and find a colleague who is free to chat/laugh/have a coffee with”*). On many occasions, the same doctor, faced with a specific difficulty, e.g., excessive numbers of visits, provided several strategies from different categories, e.g., managing visits (administrative), and communication approaches related to patients, themselves (self-knowledge), and even the team. Conversely, similar strategies could be provided for different challenges. Likewise, a single strategy, e.g., relational, could be employed to address difficulties involving certain types of patients, colleagues, or managers and administrators. Some doctors stated that they had no specific strategies for any particular difficulty. Figure 1 is a graphic illustration of the relationships between the EFPs’ ideas of what is important to be a good family physician (central circles in the diagram) and the nature of the practical strategies used: those that provide rewards at work (circles on the left) and the types of resources used to address the issues (circles on the right).

## Discussion

Although these data themselves could not be so “rich” as the narratives expected to get from other qualitative strategies, the analysis was capable of yielding meaningful qualitative insights. We also ensured rigor by analysing and presenting the results here in tandem with existing literature and conceptual frameworks. Job satisfaction for these EFP seems to be based primarily on the rewards derived, above all, appreciation of the doctors themselves, their medical, relational, and collaborative effectiveness, certain holistic and comprehensive (person-centred) practice characteristics, and a constant search for excellence. The findings clearly show that these physicians are also faced with demanding patients, insensitive managers with interests/healthcare goals they do not share, excessive paperwork, work overloading and time pressures, which are the stressors usually described in the literature and which lead to burnout of other doctors in the same setting [30–32]. To address this, our EFP foster a series of protective practices and attitudes or mindsets that coincide with those highlighted in other resilience studies [17, 33–35], and which can be included within the same domains as their sources of gratification [4]. The most significant of these are: medical, relational and collaborative effectiveness; efforts to update their knowledge; the need to preserve their personal identity by diversifying their social resources and other fields of interest; and by promoting self-knowledge and maintaining realistic expectations. The results also show a correlation between the nature of these rewarding and resilience-building elements that should be essential to a family physician and which, according to these EFP are defined by a sense of personal identity, where clinical practice takes place in the realm of relationships with the unique people they care for (with a holistic and positive sense of what health is) [36], and where aspects such as the search for excellence, self-care, teamwork, teaching and research contribute to good health to the extent that they simultaneously enrich both the doctors themselves and their patients, as well as enhancing the encounters between them. The graph in Fig. 1 highlights this correlation and proposes a hierarchical approach to these “essentials” encompassed within conceptual dimensions (central circle in the figure) of these EFP, which as a whole coincide with the fundamentals of professional identity defined by other authors [37]. The parallelism in the type of attributes identified for the three areas explored increases the coherence of these EFPs’ actions, as it demonstrates that they actually implement what they think is important. This highlights the importance of developing these core values within the profession, as a source of well-being for these EFP, but at the same time, it can be intuited that if a physician encounters difficulties developing these, this may generate suffering or “moral injury” in that doctor [20].

From this perspective, we believe that proposals to promote medical resilience can be better understood beyond simple and individual interventions, not only for physicians themselves but also in health management and medical education. Consequently, for doctors, enjoying fulfilling clinical practice and resisting its adversities is especially related to the degree to which they identify with the ultimate objectives represented by the values that define their practice, while not focusing exclusively on subordinate objectives such as making good diagnoses, publishing, earning more money, and so on. This, as some studies have pointed out [17, 35], is not only achieved by applying the traditionally proposed measures of protective practices and job gratification, but in many cases requires the doctor to develop a narrative that reflects attitudes and mentalities (mindsets) that prioritise the ultimate and integral sense of healing medicine [36] and the meaning underlying the concepts of person, encounter and relationship [37, 38]. At the employer and health administration level, measures should also be directed at changing the institutional patterns that currently appear to be locked into an increasingly complex and conflicting web of loyalties: towards patients, towards doctors, and towards the managers themselves [39], crippling the resilience of doctors and failing to recognise that caring for them leads to better patient care [21, 40]. From the “moral injury” perspective, it is clear that many physicians propose [34, 41, 42] the need to include systemic work issues that lead to more respectful treatment of doctors, suppressing autocratic mandates or policies that have a dramatic impact, offering them autonomy and authority in rational decision making, and modifying many of the current quality-of-care indicators, in addition to restoring the weight of opinion of many older physicians or those considered exemplary by their peers, such as the participants in this study. Finally, despite the fact that many educational institutions have recently incorporated this type of initiative into their curricula, with courses and specific teaching activities, it is not sufficient that this kind of action is maintained exclusively at this level. The debate here is not new and is in line with the need for medical educational establishments to profoundly overhaul their medical curricula [43, 44], to limit super-specialisation, introduce generalism, the vision of the community as a focus of work, and the socio-behavioural sciences, among other aspects, and promote selection processes that are not exclusively based on knowledge level, in order to balance the currently dominant biomedical scientific perspective [45] with a more qualitative outlook that includes broader epistemological, anthropological and ethical perspectives adapted to the reality of clinical practice [44].

### Limitations

This study has certain limitations that need to be considered. In particular, the use of an online survey, with some open-ended questions. As we mention previously, although free-text responses are not the best method to produce data rich enough to generate robust, stand-alone insights, when they meet the bar for rigorous research their analysis can generate preliminary understanding and help researchers begin to sketch content areas and to inspire new research questions [46, 47]. In order to strength the credibility and the potential of the survey results we pay special attention to some methodological aspects [46]. So, we ensure that the research questions were focused and appropriate; because the free text questions do not typically provide copious narrative feedback in the allotted space, we offered unrestricted response spaces that in many cases allowed sufficiently wide responses, also by means of a large purposive sample size we tried to ensure the sufficiency of the data in answering the research questions coherently and adequately (saturation). The pilot study allowed us both to refine the survey and the previous conceptualization of the data and their analysis. So, we carried out a thematic analysis combining a deductive and inductive approach: the first type will probably not hold for more complex answers, and the second type might end up in an unsystematic and unstructured list of categories. In the combination the development of a structured categorical system started with some themes based on the main results of the pilot study and on the theory underpinning the research project. The fine-tuning and extension that followed was text based [28]. The various backgrounds of the authors involved in the analysis (communication, physiology, statistics, and clinical practice) enriched the process of the thematic categories and subcategories, and increasing the reliability of the findings. Thus, we think the findings confirm the results of previous burnout and resilience studies, and this contributes to their explanatory power and the plausibility of the perspectives presented in this work. Nevertheless, the future research should use a less-structured qualitative approach (such as focus groups or deep interviews). On the quantitative side of the study, the number of physicians included in this purposive sample may be criticized, and particularly why using only EFPs; perhaps a more representative sample of a variety of FPs could have offered a more realistic spectrum of the values that these physicians have, however, our hypothesis considered that for a study that pretends in a preliminary way to approach the higher standards, knowing the opinion of the outstanding doctors could better reflect these standards and the deep essence of the speciality.

## Conclusions

Family doctors who are considered exemplary by their colleagues work in the same environments and encounter the same difficulties as them. These physicians, however, enjoy different aspects of their work and employ strategies to cope with challenges that are similar to those previously described in the literature. This study highlights how similar these sources of gratification and resilience strategies are to the essential attributes they prioritise for doctors practicing family medicine. This research also emphasises the central importance of these core elements as a source of well-being or potential suffering (or moral injury) for a physician and the need for doctors, managers and educators to consider them as a reference for fostering deeper and broader approaches that promote resilience and avoid burnout. However, further studies using a less structured and more comprehensive and in-depth qualitative research will be necessary to confirm and expand these results.

## Supplementary Information

**Additional file 1.** Medical Resilience and Morality SURVEY.

## Data Availability

The datasets used and analysed during the current study are available from the corresponding author on reasonable request.

## Abbreviations

EFP: Exemplary family physicians
WONCA: World Organisation of Family Doctors
FM: Family medicine
RRM: Roger Ruiz Moral
SAM: Santiago Alvarez Montero
DMM: Diana Monge Martín
CGL: Cristina García de Leonardo
