# Supplementary material for: Medical resilience and morality: a survey study on the opinions and actions of exemplary family physicians
Source: BMC Fam Pract. 2021 Oct 25;22:213. doi: 10.1186/s12875-021-01555-0 (PMC8547094; doi:10.1186/s12875-021-01555-0)
Supplement: Supplementary file 1 — Additional file 1. Medical Resilience and Morality SURVEY. [file 12875_2021_1555_MOESM1_ESM.docx]

**Additional file**

**Medical Resilience and Morality SURVEY**

1. Where do you work?

Health Centre

Urgencies / emergencies

Other (specify)

2. Years of practice

<5

6-10

11-19

19-30

> 30

3. Average number of patients per day

5-10

11-19

19-30

31-44

> 45

4. Have you got other daily work responsibilities (teaching, administrative,…)

No

Yes (detail)

5. Have you got family responsibilities?

No

Yes (detail)

6. Would you say that your job usually satisfies you professionally and personally?

Yes

No

I have not a clear idea about this

7. In relation to your work as a doctor, which of the following statements would you say best suits you?

- My work represents a burden, I suffer it daily and I do it because I have no choice.
- My work is indifferent to me and only sporadically does it give me some kind of satisfaction
- I usually enjoy my work, I usually have a good time working
- I always, or almost always, enjoy my work, it is a source of satisfaction and personal fulfillment

**ANSWER TO THIS QUESTION SECTION IF YOUR ANSWER TO QUESTION 6 WAS YES**

8. Describe between 2 and 4 aspects or reasons that you enjoy at work

9. Describe between 2 and 4 aspects that you dislike or generate dissatisfaction with your work

10. Detail some of the strategies that you usually put into practice to face (minimize or abolish) some of those aspects that you dislike and that you have already described.

11. What would you advise medical students or residents for enjoy their work as a family doctor?
